# Editorial: Host factors involved in viral infection

**DOI:** 10.3389/fmicb.2024.1382503

**Published:** 2024-03-11

**Authors:** Jose M. Jimenez-Guardeño, Luis Menéndez-Arias, Gilberto Betancor

**Affiliations:** ^1^Departamento de Microbiología, Facultad de Ciencias, Universidad de Málaga, Málaga, Spain; ^2^Instituto de Investigación Biomédica de Málaga y Plataforma en Nanomedicina-IBIMA Plataforma BIONAND, Málaga, Spain; ^3^Centro de Biología Molecular “Severo Ochoa” (Consejo Superior de Investigaciones Científicas and Universidad Autónoma de Madrid), Madrid, Spain; ^4^Instituto Universitario de Investigaciones Biomédicas y Sanitarias (IUIBS), Universidad de Las Palmas de Gran Canaria, Las Palmas de Gran Canaria, Spain

**Keywords:** virus, infection, restriction, host, antiviral

The recent severe acute respiratory syndrome coronavirus 2 (SARS-CoV-2) pandemic has brought fresh attention to viral infections. While science quickly evolves to give answers to the appearance of new viral strains, it is important that we understand the molecular mechanisms governing viral infections.

All viruses are obligated parasites, relying on cellular components to complete their replication cycle. This very feature makes them susceptible to inhibition by targeting the cellular proteins required by the virus. This is the case of cyclophilin A inhibitor cyclosporine, for treatment of influenza A virus infection, or monoclonal antibodies against anti-occludin and anti-claudin-1 for hepatitis C virus restriction. Similarly, organisms have evolved their own antiviral mechanisms, best exemplified by the adaptative immune response mediated by antibodies. Another key example is the production of antiviral factors. These are proteins able to inhibit infection by targeting viral proteins or processes. Many of these are interferon-stimulated genes (ISGs) with broad antiviral activity, such as MX1, MX2, or ISG15. Nevertheless, viruses have evolved countermeasures to inactivate major restriction factors, as is the case of HIV-1 Vpu and tetherin (BST-2), or to directly inhibit the interferon response, as is the case of SARS-CoV-2 protein ORF6.

This delicate balance between infection/restriction has marked the evolution of all viruses, which constantly need to fabricate new ways of modulating the cell environment to complete their life cycle. In other words, and as the Red Queen said to Alice in Lewis Carroll's *Through the Looking Glass*, “it takes all the running you can do, to keep in the same place.”

This Research Topic aimed to contribute to our understanding of the intricate interplay between viruses and cells. The publication of eight articles has undeniably shed light on certain unknown aspects of this complex interaction, offering valuable insights and answers.

Viljakainen et al., studied the presence of viral infections in three ant species, finding a striking host-specificity of the viruses identified. Only one of the 59 viruses identified was shared by more than one ant species. In addition, their work determined a host ant-specific RNAi response (21 vs. 22 nt siRNAs) in the different ant species. By differentiating infections causing disease from those that do not, competent bodies could better manage emergent infections in the ecosystem.

Liang et al., provide valuable information on Echovirus 30 infection. This virus has been recently identified as a causative agent of viral encephalitis and meningitis in children. Therefore, understanding the molecular and cellular mechanisms underpinning the Echovirus 30 life cycle is pivotal for the development of therapeutic strategies.

Ohta et al., present data demonstrating how Hazara orthonairovirus, a representative of the pathogenic *Nairoviridae* family, downregulates the expression of claudin-1 by means of its nucleoprotein. This allows the virus to spread the infection by overcoming the barrier induced by tight junctions in epithelial cells. This article presents another example of how cell restriction factors are counteracted by viral proteins.

Devaux and Fantini proposed a novel model, which the authors coined as “boomerang effect” to explain how rare ACE2 alleles present in certain human populations may have higher affinity for the spike protein of specific SARS-CoV-2 variants. This could have provided an evolutionary pressure on the virus to develop higher-affinity spikes directed to these rare ACE2 mutants. With most SARS-CoV-2 vaccines directed against the receptor-binding domain of the spike viral protein, this phenomenon could indeed impact the humoral response against the virus.

Ma et al., provide data supporting a novel model where herpes simplex virus 1 (HSV-1) promotes the packaging of the transcription factor octamer-binding transcription factor 1 (Oct-1) into extracellular vesicles in infected cells. By inducing the synthesis of these vesicles, HSV-1 ensures sizeable amounts of Oct-1 are being transported to the extracellular space. This Oct-1 incorporated in *trans* by not-infected cells prime them for infection not only by HSV-1, but for other RNA viruses as well. This article shows how infection by one virus can alter the environment producing an unspecific effect that can be exploited by other viruses.

Kang et al., studied one of the most versatile ISGs, ISG15. Interestingly, the authors demonstrate that opposite to the effect ISG15 normally exerts on many viral families, ovine ISG15 (oISG15) promotes the infection of the Sedoreovirus Bluetongue virus. The authors propose that the interaction of oISG15 with viral proteins prevents the autophagy-dependent degradation of viral NS1, resulting in enhanced viral infection. This study provides an attractive example of how the race between viral evolution and cellular restriction can turn a common restriction factor into a virus cofactor.

Zhang et al., present a thorough review of the role Seneca valley virus (SVV) 3C protease plays in the regulation of the innate immune response of the infected cell. The innate immune response is the first line of defense against viral infection, making it a common target of viral proteins, which actively seek to disable this vital defense mechanism. Understanding the various inhibitory mechanisms employed by different virus families is of great importance for the control and prevention of new infections.

Wang et al., present a review analyzing one of the least studied mechanisms by which cells fight back virus infection. Therefore, the authors elegantly summarize the state-of-the-art research supporting the role of ferroptosis as a regulated cell death (RCD) mechanism involved in the replication of different pathogens. This review is of interest not only to virologists but also to a broader audience interested in the phenomenon of ferroptosis, including iron metabolism, lipid peroxidation, and antioxidant metabolism.

Collectively, these papers are representative of current efforts to understand virus-host interactions and the role of host factors in viral infection. We hope that this Research Topic will contribute to fostering renewed interest in the field.

## Author contributions

JJ-G: Conceptualization, Writing – review & editing. LM-A: Conceptualization, Writing – review & editing. GB: Conceptualization, Writing – review & editing, Project administration, Supervision, Writing – original draft.

